# Framework for field-scale application of molecular biological tools to support natural and enhanced bioremediation

**DOI:** 10.3389/fmicb.2022.958742

**Published:** 2022-11-08

**Authors:** Trent A. Key, Skyler J. Sorsby, Yingnan Wang, Andrew S. Madison

**Affiliations:** ^1^ExxonMobil Environmental and Property Solutions Company, Spring, TX, United States; ^2^Golder Associates USA Inc., Marlton, NJ, United States; ^3^Imperial Oil Limited, Calgary, AB, Canada

**Keywords:** molecular biological tools, bioremediation, biodegradation, natural attenuation, enhanced bioremediation, groundwater, contaminated site management

## Abstract

Microorganisms naturally present at environmental contaminated sites are capable of biodegrading, biotransforming, or removing contaminants in soil and groundwater through bioremediation processes. Cleanup strategies and goals for site remediation can be effectively achieved by bioremediation leveraging the capabilities of microorganisms to biotransform contaminants into lesser or non-toxic end products; however, reproducible success can be limited by inadequate design or performance monitoring. A group of biological analyses collectively termed molecular biological tools (MBTs) can be used to assess the contaminant-degrading capabilities and activities of microorganisms present in the environment and appropriately implement bioremediation approaches. While successful bioremediation has been demonstrated through previously described lab-scale studies and field-scale implementation for a variety of environmental contaminants, design and performance monitoring of bioremediation has often been limited to inferring biodegradation potential, occurrence, and pathways based on site geochemistry or lab-scale studies. Potential field-scale application of MBTs presents the opportunity to more precisely design and monitor site-specific bioremediation approaches. To promote standardization and successful implementation of bioremediation, a framework for field-scale application of MBTs within a multiple lines of evidence (MLOE) approach is presented. The framework consists of three stages: (i) “Assessment” to evaluate naturally occurring biogeochemical conditions and screen for potential applicability of bioremediation, (ii) “Design” to define a site-specific bioremediation approach and inform amendment selection, and (iii) “Performance Monitoring” to generate data to measure or infer bioremediation progress following implementation. This framework is introduced to synthesize the complexities of environmental microbiology and guide field-scale application of MBTs to assess bioremediation potential and inform site decision-making.

## Introduction

There is growing interest in applying natural approaches to contaminated site management, such as bioremediation which may have lower energy or resource consumption as compared to mechanical or chemical remediation approaches (USEPA, 2008; Huang et al., 2016). Bioremediation utilizing native microorganisms capable of biodegrading, biotransforming or removing contaminants in soil and groundwater (e.g., petroleum hydrocarbons, halogenated organics, cyclic ethers, inorganics) has been demonstrated to successfully achieve site remediation as a standalone remedial technology or in combination with other remedial technologies (Bouwer and Zehnder, 1993; Wiedemeier et al., 1999; Bombach et al., 2010). Despite increased application of bioremediation and increased understanding of the influence biological processes have on the environmental fate of contaminants, greater emphasis is often focused on evaluations of physicochemical attenuation processes (e.g., sorption, desorption, volatilization, dilution, diffusion, and advection) to inform remediation strategies. When biogeochemical attenuation processes are evaluated, contaminant biodegradation is often inferred based on a limited suite of biogeochemical parameters (Wiedemeier et al., 1999; Rittmann and McCarty, 2001; Lawson et al., 2019). This approach can result in an incomplete and underestimated role of biodegradation and can propagate uncertainty in the conceptual site model (CSM) and potential implementation of ineffective remediation strategies.

During the past 20 years, molecular biological tools (MBTs) have been increasingly utilized due to scientific advancements and decreased analytical costs, to directly assess microbiological processes at environmental sites (Wilson et al., 1999, 2019; Madsen, 2000; Beller et al., 2002; Winderl et al., 2007; Cupples, 2008; Gedalanga et al., 2016; Taggart and Clark, 2021; Adamson et al., 2022). MBTs, such as quantitative polymerase chain reaction (qPCR), can be used to directly assess abundance of contaminant-degrading microorganisms or functional genes which encode for contaminant-degrading enzymes present in the environment. Increased, appropriate application of MBTs can shift the historical site assessment paradigm to further increase knowledge of field-scale microbiological processes, improve bioremediation approaches to be more precisely engineered, and ultimately promote bioremediation success and eventual site closure.

While prior publications have focused on the advantages of employing MBTs in concert with contaminant chemistry and geochemistry evaluations to reduce site uncertainties and better characterize subsurface microbiology (Rittmann and McCarty, 2001; Amos et al., 2008; Busch-Harris et al., 2008; Bombach et al., 2010; Lebron et al., 2015; Gedalanga et al., 2016; Zhang et al., 2016; Bouchard et al., 2018; Lawson et al., 2019; Wilson et al., 2019; Taggart and Clark, 2021; Adamson et al., 2022), it has been observed that application, implementation strategies, and data interpretation of MBTs at the field-scale are inconsistent in practice and could potentially become barriers to uptake and acceptance within the contaminated site management community.

In this perspective, a framework is introduced to synthesize the complexities of environmental microbiology and guide field-scale application of MBTs to assess bioremediation potential and inform site decision-making. This framework consists of following a staged process and a multiple lines of evidence (MLOE) approach to meet site-specific objectives associated with contaminant biodegradation or biotransformation using field-scale data. Example application of this framework can be found in another article in this research topic issue (Madison et al., in review).

## Molecular biological tools to assess bioremediation

**FIGURE 1:**
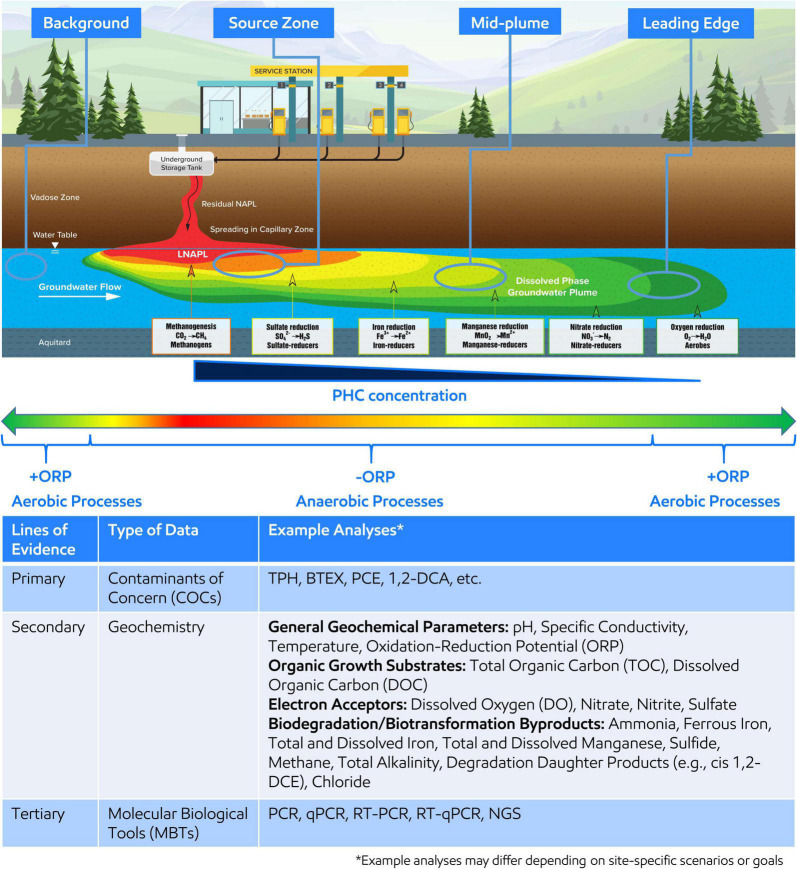
**(Top)** Idealized groundwater plume resulting from petroleum hydrocarbon (PHC) release and the dominant redox conditions, electron acceptor utilized during microbial respiration, and associated microorganisms along the plume length. Oxidative reduction potential (ORP) is shown across the plume length where red, orange, yellow, and yellow-green indicate reducing conditions associated with the highest PHC concentration around the light non-aqueous phase liquid (LNAPL) body or source zone and green indicates oxidizing conditions associated with the lowest PHC concentration at the leading edge of the plume or upgradient of the plume. Four areas of the plume, background, source zone, mid-plume, and leading edge, are circled to indicate recommended areas for MBT sampling. **(Bottom)** The table demonstrates and summarizes example analyses to be considered when developing an MBT sampling plan within a MLOE approach.

With little exception, microorganisms are ubiquitous within the subsurface of environmental contaminated sites (Bouwer and Zehnder, 1993; Wiedemeier et al., 1999; Rittmann and McCarty, 2001). Microorganisms gain energy by catalyzing biochemical reactions that involve breaking chemical bonds and transferring electrons from organic carbon sources, such as including natural organic matter or contaminants such as petroleum hydrocarbons (PHCs), or inorganic electron donors (e.g., H_2_) (Bouwer and Zehnder, 1993; Wiedemeier et al., 1999; Chapelle, 2000; Christensen et al., 2001; Löffler et al., 2013). The electrons are transferred to several electron acceptors naturally present in the subsurface, such as nitrate (NO_3_^–^), oxidized manganese (Mn^3+/4+^), oxidized iron (Fe^3+^), sulfate (SO_4_^2–^), and carbon dioxide (CO_2_). Since utilizing different electron acceptors yield varying energies (i.e., Gibbs free energy yield), microorganisms prefer to utilize the electron acceptor with the highest energy gain first resulting in a preferential use sequence of O_2_ > NO_3_^–^ > Mn^3+/4+^ > Fe^3+^ > SO_4_^2–^ > CO_2_ (Bethke et al., 2011). A schematic of the resulting idealized plume from a PHC spill is shown on Figure 1, which is annotated with the distribution of typical electron acceptor utilization and associated microorganisms.

At the time of writing this perspective, commercially available and scalable MBTs which can be more readily applied at field-scale to support site management decision-making within a MLOE approach are nucleic acid-based analyses that include qPCR, reverse transcriptase qPCR (RT-qPCR), and next generation sequencing (NGS) of the 16S rRNA gene.

qPCR can be used to detect and quantify microbial genes within environmental samples. It should be noted that genes detected and measured within environmental samples could be from both live- and dead-cells, which should be considered during data interpretation. Target genes include functional genes that encode enzymes implicated in contaminant biodegradation, such as benzoyl-coenzyme A reductase (BCR) which reduces benzoyl-coenzyme A (an intermediate in anaerobic toluene, phenol, and ethylbenzene biodegradation) (Boll and Fuchs, 1995), key functions of target microorganisms such as the adenosine-5’-phosphosulfate reductase (APR) which synthesizes sulfite in dissimilatory sulfate reduction (Ramos et al., 2012), or 16S rRNA genes for specific contaminant-degrading taxa such as *Dehalogenimonas* spp. (Chen et al., 2014). The presence of target genes or microorganisms at abundances above background can provide a tertiary line of evidence of biodegradation potential to support the contaminant chemistry and geochemistry data.

RT-qPCR is a variation of qPCR, which can be used to quantify gene expression [i.e., messenger ribonucleic acids (mRNA)] indicative of biological activity. The occurrence of mRNA expressed from genes linked to biodegradation indicates that the associated contaminant-degrading microorganisms are metabolically active and carrying out biodegradation processes (Wilson et al., 1999; Winderl et al., 2007; Bombach et al., 2010; Key et al., 2014; Bouchard et al., 2018; Lawson et al., 2019). While positive detections of mRNA transcripts in environmental samples can provide strong evidence of biodegradation occurrence, the absence of specific mRNA transcripts should not be interpreted to indicate that biodegradation activities are not occurring. The abundance of mRNA transcripts in environmental samples may be low and mRNA transcripts are generally short-lived (Wilson et al., 1999; Interstate Technology and Regulatory Council, 2013; Bouchard et al., 2018). Consequently, mRNA transcripts may not be detected although biodegradation is occurring (Bouchard et al., 2018).

NGS of the 16S rRNA gene can be used to identify microorganisms in a sample and assess the composition of the microbial community. The 16S rRNA gene is evaluated to identify and assess prokaryotes (bacteria and archaea) at contaminated sites. NGS can most relevantly be used when gene targets linked to contaminant biodegradation (i.e., functional genes) are not known, and to further screen trends in community composition to identify genera or species with potential contaminant-degrading capabilities (Hidalgo et al., 2020). These data can be evaluated to understand how microbial community composition change associated with contaminant or geochemical concentrations, spatially across a site, over time, or during remediation.

In addition to these nucleic acid-based MBTs, there are other approaches that can be leveraged to measure or infer contaminant biodegradation, such as proteomics, metabolomics, and isotope-based methods. While proteomics and metabolomics measuring enzymes or intermediates are less often currently used at contaminated sites, expanded use of these MBTs in the future is anticipated. Further, isotope-based methods, such as compound specific isotope analysis (CSIA), are currently considerably used at contaminated sites and can be considered where applicable (Bouchard et al., 2018; Phillips et al., 2019).

## Framework

**FIGURE 2:**
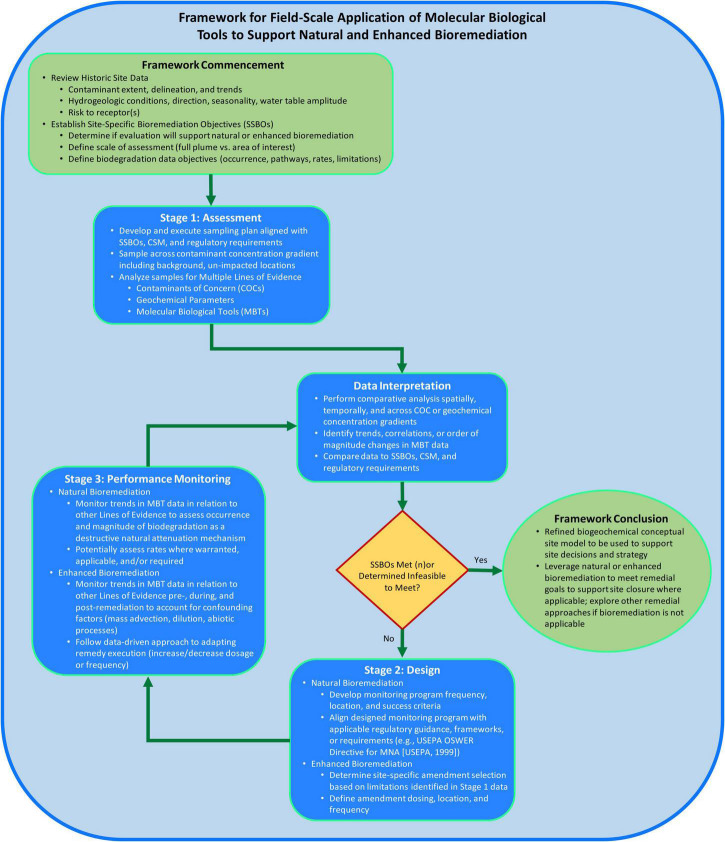
Framework for field-scale application of molecular biological tools (MBTs) to support bioremediation process flow diagram consisting of logic and considerations for when, where, why, and how to apply MBTs at the field-scale to support site decision-making.

The framework structure (Figure 2) proposed herein consists of two elements to support consistent application of MBTs at the field-scale and to increase likelihood of successful bioremediation implementation. The two elements are: (1) following a staged process and (2) using a MLOE approach for data generation and interpretation to address sites-specific bioremediation objectives (SSBOs). The proposed framework builds upon the approach of previously published documents describing how to measure, observe, or execute monitored natural attenuation (MNA) or enhanced bioremediation (USEPA, 1999; Lebron et al., 2015; Wilson et al., 2019; Taggart and Clark, 2021; Adamson et al., 2022). The objective of the framework is to provide a systematic approach to assess applicability of bioremediation, support CSM development by measuring biogeochemical processes, design a bioremediation approach, and monitor bioremediation performance using field-scale MBT data.

### Framework commencement

At the onset of applying this framework, historical site data should be reviewed to understand the nature, extent, trends of contamination and associated site risk-drivers (Figure 2). Hydrogeological information should be reviewed to inform understanding of the groundwater flow direction(s) and gradient. Existing chemical contaminant data should be reviewed to identify contaminant(s) of interest, impacted media, determine the vertical and lateral extent of impacts, and characterization of contaminant fate and transport through spatial and temporal concentration trends. Overall, the conclusions of these data evaluations should be integrated into a coherent CSM describing source(s), contaminant fate and transport processes, and receptor exposure to inform the need for and scope of generating MBT data to support bioremediation. Lastly, SSBOs should be defined to drive focus for the intent of MBT data generation, such as determining whether data will be used to support a natural or enhanced bioremediation strategy and develop biodegradation data objectives (i.e., assess occurrence vs. rates).

### Stage 1: Assessment

The objective of the assessment stage is to identify potential biodegradation or biotransformation processes to inform the suitability of bioremediation. The assessment stage is where field-scale MBT data may be first generated to develop or refine the site biogeochemical CSM. While the assessment stage within this framework is not to be confused with project site characterization and is applicable across the project lifecycle where bioremediation may be considered (e.g., remedy selection of bioremediation instead of pump and treat), the generation of MBT data should be done intentionally to support answering site-specific questions or achieving site-specific objectives (i.e., SSBOs).

A sampling plan should be developed and implemented in alignment with SSBOs, CSM, and regulatory requirements. Samples should be collected at target locations across the area(s) of interest related to contaminant(s) concentration and geochemical gradients and include unimpacted background location(s) (Figure 1). The goal of targeted sampling across chemical and geochemical gradients is to capture associated microbial changes to support comparative analysis and interpretation. Further, understanding of the distribution of electron acceptors, general biogeochemical parameters, and contaminants across the area of interest supports characterization of subsurface microbial processes and should also be concurrently analyzed as part of the MLOE approach—Figure 1 (Bottom) describes an example analyte list (Wiedemeier et al., 1999; Chapelle, 2000; Christensen et al., 2001). qPCR analysis for functional or taxonomic genes (e.g., benzylsuccinate synthase or 16S rRNA gene) is often employed in Stage 1 and gene abundance (or presence/absence) are correlated to contaminant concentration or geochemical gradients.

Subsequently generated contaminant, geochemical, and MBT data can be interpreted through spatial and/or temporal comparative analysis to support determination of bioremediation potential. MBT data interpretation should be aligned with previously developed MLOE approaches and/or MNA guidance, and should consist of assessing contaminant trends for direct or indirect evidence of biodegradation, geochemical conditions to identify biodegradation mechanisms and potential electron acceptor/donor limitations of biological activity, presence and abundance of contaminant-degrading microorganisms or genes, and the inferred extent of intrinsic biodegradation (USEPA, 1999; Wilson et al., 2019; Taggart and Clark, 2021; Adamson et al., 2022). This approach is demonstrated by Baldwin et al. (2008) where benzene, toluene, ethylbenzene and xylenes (BTEX) concentrations were correlated with aromatic oxygenase genes to assess bioremediation potential. These data supported determination that an intrinsic bioremediation approach (i.e., natural attenuation) was feasible across large plume areas and that an enhanced bioremediation approach was required in the smaller portions of the plumes where BTEX levels were increasing. For site areas where data interpretation suggests enhanced bioremediation is valuable, data should be reviewed to identify current redox conditions, microbial processes, and potential biodegradation limitations to support selection of a site-specific amendment (e.g., pH buffer, sulfate, fermentable substrate). Lastly, all data should be compared to SSBOs, CSM, and regulatory requirements, and where SSBOs are met or determined infeasible to meet, no further progression of the framework is warranted (e.g., Framework Conclusion).

### Stage 2: Design

If the results of Stage 1 suggest SSBOs have not been met nor determined infeasible to meet, the framework proceeds into the design stage (Stage 2). When a natural bioremediation strategy is posited to accomplish site remediation objectives, the generated contaminant, geochemical, and MBT datasets from Stage 1 should provide sufficient, conclusive evidence to support occurrence of intrinsic bioremediation across the area of interest. In addition to demonstrating natural bioremediation, the Stage 1 MLOE datasets should guide the monitoring program design including selection of sample locations (range of contaminant and geochemical conditions including background) and sampling parameters to assess occurrence of natural bioremediation as a mechanism of natural attenuation.

When an enhanced bioremediation remedy is being evaluated and designed, Stage 1 data and observations should be used to support the determination of biogeochemical processes and limitations important to design a site-specific enhanced bioremediation. During the design stage, Stage 1 data should guide site-specific enhanced bioremediation approaches, such as adding a site-specific amendment for biostimulation {e.g., fermentable substrate or electron donor [emulsified vegetable oil (EVO), lactate, formate], electron acceptor [oxygen, nitrate, sulfate], nutrients [nitrogen, phosphorous, vitamins], pH, temperature}, and/or microbial cultures (e.g., bioaugmentation). Additionally, Stage 1 data should be used to support the estimation of amendment dosing, location and frequency based on observed biogeochemical processes and stoichiometry. Field-scale pilot testing is recommended to be performed to refine amendment dosing and frequency, and field-scale feasibility and effectiveness prior to full-scale implementation. Geochemical parameters are utilized to monitor the biogeochemical effects of the approach while qPCR or other MBTs monitor the effects on the microbial population in response to biostimulation or bioaugmentation. This approach was employed in a pilot and full-scale remedial application to demonstrate organic carbon amendment addition promoted growth of *Dehalococcoides* spp. and stimulated complete reductive dechlorination of tetrachloroethene (PCE), trichloroethene (TCE) and daughter products cis-1,2-dichloroethene and vinyl chloride to non-toxic end product ethene (Davis et al., 2008).

### Stage 3: Performance monitoring

Performance monitoring (Stage 3) follows design and initial implementation of the natural or enhanced bioremediation strategy. While contaminant concentrations ultimately must show progress toward the remedial goals, sampling and analysis of select geochemical parameters and MBTs during remedy performance monitoring can improve understanding of remedy progress. MBT application may consist of qPCR analyses to assess whether contaminant-degrading microorganisms are sufficiently abundant to sustain desired biodegradation. Temporal and spatial trends for qPCR results can be particularly helpful to demonstrate positive, negligible, or negative effects on microbial processes to refine the remedial strategy across the treatment area, and in some cases serve as a leading indicator of remedy performance. Contaminant concentrations can, in some cases, be a lagging indicator of enhanced bioremediation due to various fate and transport mechanisms which may act as confounding factors impeding the ability to distinguish occurrence or enhancement of contaminant biodegradation. For example, as the rates of contaminant biodegradation are increased due to successful enhanced bioremediation, the rate of dissolution of residual non-aqueous phase liquids (NAPL) may also increase to maintain multi-phase equilibrium (Seagren et al., 1994; Amos et al., 2008, 2009). Additionally, RT-qPCR can be employed if there is a need to verify expression of genes encoding for enzymes involved in contaminant biodegradation.

Data generated in Stage 3 should be continually reviewed to assess remedy performance monitoring. Data interpretation should be done as described in section “Stage 1: Assessment,” consisting of comparative analysis across contaminant and geochemical concentration gradients, within a MLOE approach, and compared to SSBOs, CSM, and regulatory requirements. In a study where sulfate was introduced to the subsurface, an integrated performance monitoring program of contaminants, geochemical parameters, and MBTs demonstrated that sulfate injection increased the rates of biodegradation of BTEX (Sublette et al., 2006). As sulfate was utilized as an electron acceptor, the subsurface microbial community became more anaerobic and abundance of the *bssA* gene (involved in the first step of anaerobic degradation of toluene and xylene; Beller et al., 2002) increased.

### Framework conclusion

At the conclusion of the framework, whether having progressed through all three stages or solely Stage 1, a biogeochemical CSM should have been developed or refined to support subsequent site decision-making and strategy. Microbiology plays an important role in contaminant fate and attenuation, but is often times unmeasured, inferred, or not considered at contaminated sites. Applying this framework will aid in improving knowledge of microbial processes and broader contaminant fate at contaminated sites. Additionally, data-driven decisions based on this framework should improve the effectiveness of natural or enhanced bioremediation to meet site goals and achieve or progress toward site closure, as well as avoid the application of bioremediation where it may be infeasible.

## Discussion

Bioremediation methods have been demonstrated to be favorable strategies for contaminated site management to naturally biodegrade, biotransform or remove contaminants in soil and groundwater. As discussed in this perspective, the increasing application of MBTs to directly measure microbiological processes at environmental sites has the potential to reduce uncertainty related to site-specific biogeochemical processes. However, field-scale MBT application, implementation strategies, and interpretation can be inconsistent across varying sectors, institutions, or individuals potentially posing barriers to increased uptake and acceptance. For this reason, the field-focused framework described herein was developed to synthesize complexities of environmental microbiology and assess bioremediation potential at the field-scale during various stages of planning and execution.

Application of this framework should be considered for use at contaminated sites implementing bioremediation; further description of application can be found in another article in this research topic issue (Madison et al., in review). It should be noted that this framework is limited in its applicability to contaminants which have inadequate knowledge on biodegradation mechanisms or undeveloped associated MBTs. The value of following the suggested standardized approach is to potentially reduce uncertainty related to biogeochemical processes and contaminated fate, and increase the likelihood of successful implementation of bioremediation approaches. Future efforts should explore the potential for systematic data transformation of field-scale MBT data into biodegradation rates.

## Data availability statement

The original contributions presented in this study are included in the article/supplementary material, further inquiries can be directed to the corresponding author.

## Author contributions

TK and AM contributed to the conception and design of this perspective. SS and YW refined and sharpened conceptual layout and messaging of content. TK, SS, and AM wrote sections of the manuscript. All authors contributed to the manuscript review, read, and approved the submitted manuscript.
